# The Diagnostic Value of MRI-Based Texture Analysis in Discrimination of Tumors Located in Posterior Fossa: A Preliminary Study

**DOI:** 10.3389/fnins.2019.01113

**Published:** 2019-10-23

**Authors:** Yang Zhang, Chaoyue Chen, Zerong Tian, Ridong Feng, Yangfan Cheng, Jianguo Xu

**Affiliations:** ^1^Department of Neurosurgery, West China Hospital, Sichuan University, Chengdu, China; ^2^West China School of Medicine, West China Hospital, Sichuan University, Chengdu, China

**Keywords:** magnetic resonance imaging, texture analysis, posterior fossa tumors, medulloblastoma, brain metastatic tumor, hemangioblastoma

## Abstract

**Objectives:**

To investigate the diagnostic value of MRI-based texture analysis in discriminating common posterior fossa tumors, including medulloblastoma, brain metastatic tumor, and hemangioblastoma.

**Methods:**

A total number of 185 patients were enrolled in the current study: 63 of them were diagnosed with medulloblastoma, 56 were diagnosed with brain metastatic tumor, and 66 were diagnosed with hemangioblastoma. Texture features were extracted from contrast-enhanced T1-weighted (T1C) images and fluid-attenuation inversion recovery (FLAIR) images within two matrixes. Mann–Whitney *U* test was conducted to identify whether texture features were significantly different among subtypes of tumors. Logistic regression analysis was performed to assess if they could be taken as independent predictors and to establish the integrated models. Receiver operating characteristic analysis was conducted to evaluate their performances in discrimination.

**Results:**

There were texture features from both T1C images and FLAIR images found to be significantly different among the three types of tumors. The integrated model represented that the promising diagnostic performance of texture analysis depended on a series of features rather than a single feature. Moreover, the predictive model that combined texture features and clinical feature implied feasible performance in prediction with an accuracy of 0.80.

**Conclusion:**

MRI-based texture analysis could potentially be served as a radiological method in discrimination of common tumors located in posterior fossa.

## Introduction

Medulloblastoma, brain metastatic tumor, and hemangioblastoma are the three types of tumors with highest incidents located in posterior fossa; among these, medulloblastoma mostly occurs in pediatric population (Brandao and Young Poussaint, 2017; D’Arco et al., 2018), while hemangioblastoma and brain metastatic tumor are two of the most common posterior fossa tumors in adult population (Shih and Smirniotopoulos, 2016). Magnetic resonance (MR) scan has been considered as the most practical examination in diagnosis of intracranial tumors for clinicians (Poretti et al., 2012; Plaza et al., 2013). Typically, the image of medulloblastoma in enhanced pattern appears as a heterogeneous mass with different degrees of enhancement; brain metastatic tumor appears as peritumoral edema and avid enhancement; and hemangioblastoma appears as a cystic mass with the enhancing mural nodule (Plaza et al., 2013; Shih and Smirniotopoulos, 2016). However, the radiological diagnostic accuracy largely depends on the judgment and expertise of radiologists. Besides, brain metastatic tumor may present various MR image features based on their primary origins, and the various appearances of medulloblastoma and hemangioblastoma could potentially lead to misleading conclusions in some cases (Poretti et al., 2012; Millard and De Braganca, 2016; Payabvash et al., 2018). However, it is important to make preoperative diagnosis accurately and early as the treatment strategies and prognoses of patients are dramatically different (Grossman and Ram, 2016; Kang et al., 2017).

Texture analysis is the radiomic method that could extract mathematically defined features from diverse medical images and provide quantitative information beyond human eye assessment. In previous studies, texture analysis has been applied as a radiological tool in differentiation, treatment monitoring, and prognosis prediction of various types of tumors (Ion-Margineanu et al., 2016; Jalil et al., 2017; Kim et al., 2017; Chaddad et al., 2018; Lisson et al., 2018; Giannini et al., 2019). However, there has been few researches to explore its value in discrimination of posterior fossa tumors. In the present study, we performed analyses to investigate the ability of texture analysis on conventional magnetic resonance imaging (MRI) in discriminating the three most common posterior fossa tumors: medulloblastoma, brain metastatic tumor, and hemangioblastoma. The diagnostic performances were evaluated with establishment of radiomic parameters and integrated model to evaluate their diagnostic values.

## Materials and Methods

### Patient Selection

All patients involved in this retrospective study were diagnosed and treated at the neurosurgery department of our institution from March 2015 to July 2018. We initially screened the database of our institution to select the potentially eligible patients who were (1) with pathological confirmation (on medulloblastoma or brain metastatic tumor or hemangioblastoma), (2) with available preoperative high-quality MR images, and (3) with complete and elaborate electronical medical records. Then, the patients were excluded if they had the history of any other cerebral diseases. Clinical characteristics of qualified patients were recorded, and written informed consent was obtained from all participants included in the study. For patients under the age of 16, written informed consent was obtained from their parents or guardians. This study was approved by the Ethics Committee of Sichuan University.

### MRI Acquisition

Magnetic resonance scans were performed in the MR Research Center of our institution using 3.0 T Siemens Trio Scanner. The sequences included conventional T1-weighted images, contrast-enhanced T1-weighted (T1C) images, T2-weighted images, and fluid-attenuation inversion recovery (FLAIR) images, acquiring axial, coronal, and sagittal data. The scanning of T1C images was performed within 180 s after injection of gadopentetate dimeglumine (0.1 mmol/kg) as the contrast agent. The parameters were as follows: time repetition = 2000 ms, time echo = 30 ms, voxel size = 3.75 mm^3^ × 3.75 mm^3^ × 5 mm^3^, flip angle = 90°, slice thickness = 5 mm, matrix = 64 × 64, and field of view = 240 mm^2^ × 240 mm^2^. The preoperative MR images of all participants and their radiological reports were collected from our institutional radiology department.

### Texture Features Extraction

**FIGURE 1:**
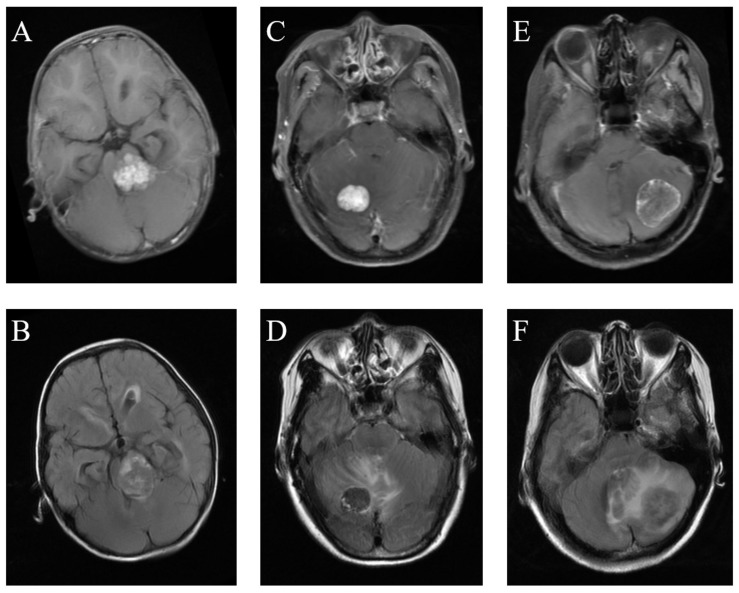
Comparison of MRI among medulloblastoma, hemangioblastoma, and brain metastatic tumor. **(A)** Medulloblastoma on T1C images. **(B)** Medulloblastoma on FLAIR images. **(C)** Hemangioblastoma on T1C images. **(D)** Hemangioblastoma on FLAIR images. **(E)** Brain metastatic tumor on T1C images. **(F)** Brain metastatic tumor on FLAIR images.

**FIGURE 2:**
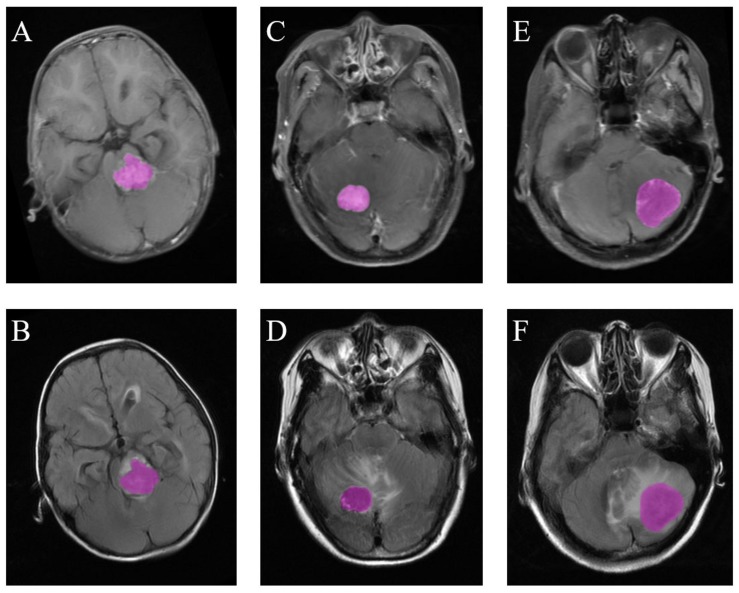
Examples of ROI delineation in the three types of tumors. **(A)** Medulloblastoma on T1C images. **(B)** Medulloblastoma on FLAIR images. **(C)** Hemangioblastoma on T1C images. **(D)** Hemangioblastoma on FLAIR images. **(E)** Brain metastatic tumor on T1C images. **(F)** Brain metastatic tumor on FLAIR images.

The extraction of texture features was performed by two neurosurgeons together using LifeX package^1^ with the assistance of senior radiologists (Nioche et al., 2018). T1C images and FLAIR images were selected to conduct texture analysis, considering their rather clear depictions on the border of tumors compared with other sequences like conventional T1-weighted images and T2-weighted images (Figures 1, 2). Two neurosurgeons manually drew along the border of tumors to obtain regions of interest (ROI) on the axial image since it was relatively accurate for ROI delineation on this view. After continuously contouring the tumor on each layer, three-dimensional texture features could be automatically calculated by the software with default setting. Disagreements on the boundary of the tumor were recorded and addressed by consulting the senior radiologists or senior neurosurgeons. Based on previous studies, a total of 10 texture features from two representative matrixes were selected, including Energy, Entropy, Kurtosis, Skewness derived from histogram-based matrix (HISTO), and Correlation, Contrast, Dissimilarity, Energy, Entropy, Homogeneity derived from gray-level co-occurrence matrix (GLCM) (Rodriguez Gutierrez et al., 2014; Lakhman et al., 2017; Fujima et al., 2019; Yin et al., 2019). The definitions of these features were as follows: HISTO-Energy measures the uniformity of the distribution; HISTO-Entropy measures the randomness of the distribution; HISTO-Kurtosis measures whether the gray-level distribution is peaked or flat relative to a normal distribution; HISTO-Skewness measures the asymmetry of the gray-level distribution in the histogram; GLCM-Correlation reflects linear dependency of gray levels in GLCM; GLCM-Contrast reflects local variations in the GLCM; GLCM-Dissimilarity reflects variation of gray-level voxel pairs; GLCM-Energy reflects uniformity of gray-level voxel pairs; GLCM-Entropy reflects randomness of gray-level voxel pairs; and GLCM-Homogeneity reflects homogeneity of gray-level voxel pairs (Nardone et al., 2018).

### Statistical Analysis

Pairwise comparisons were firstly performed to evaluate the statistical differences with the Mann–Whitney *U* test among the three types of tumors: part one (medulloblastoma vs. hemangioblastoma), part two (medulloblastoma vs. brain metastatic tumor), and part three (brain metastatic tumor vs. hemangioblastoma). Binary logistic regression analysis was performed with the standardized features to evaluate if they could be taken as independent predictors, while variance inflation factor (VIF) among different texture features was assessed first to avoid the interference of collinearity. Moreover, the independent predictors were integrated as Z score based on the following formula:

Z=∑i=1n(Bi⁢Xi*)

(*X*_i_ indicated each independent texture feature, and *B*_i_ was the regression coefficient of each feature). Receiver operating characteristic (ROC) analysis was performed on both independent predictors and integrated *Z* score to evaluate their practical values in discrimination. Area under the curve (AUC), 95% confidence interval (CI), optimal cutoff values (at the maximal Youden’s index), sensitivity, specificity, and standard error were recorded. Then, texture features were assessed together using Kruskal–Wallis *H* test and multinomial logistic regression. Significant texture features, as well as age (recognized clinical discriminative parameter), were included in multinomial logistic regression to build the predictive model for the three types of tumors.

In order to compare the discriminative performance between radiomics-based models and radiologists, we recorded the primary diagnosis of preoperative MRI on radiological reports as the judgment of radiologists and compared its accuracy with our predictive models. The statistics were considered significant if the *p* value < 0.05. All statistical analyses were performed with IBM SPSS Statistics for Windows (Version 22.0, IBM Corp., Armonk, NY, United States) and MedCalc statistics (MedCalc Software, Belgium).

## Results

### Characteristics of Patients

A total number of 185 eligible patients were selected from our institutional database. Sixty-six of them were diagnosed with hemangioblastoma, 63 patients were diagnosed with medulloblastoma, and 56 patients were diagnosed with brain metastatic tumor. Among the brain metastatic tumors, 32 originated from lung cancer, 16 originated from breast cancer, while 8 originated from other types of tumors, including melanoma, renal cell carcinoma, colon cancer, and Wilm’s tumor. The gender ratio was 1.52 (male: 112, female: 73). Mean age of patients with medulloblastoma was 9.06 ± 7.61 years, brain metastatic tumor was 57.61 ± 12.57 years, and hemangioblastoma was 45.12 ± 17.47 years. T1C images were available for all patients, while FLAIR images were available for 156 patients. The detailed characteristics of the patients were summarized in Table 1.

**TABLE 1:** Characteristics of patients.

**Characteristics**	**Medulloblastoma**	**Brain metastatic tumor**	**Hemangioblastoma**
Number	63	56	66
**Gender**			
Male	42	36	34
Female	21	20	32
**Age (year)**			
Mean ± SD	9.06 ± 7.61	57.61 ± 12.57	45.12 ± 17.47
Range	1–37	12–85	11–79

*SD, standard deviation.*

### Part 1 (Medulloblastoma vs. Hemangioblastoma)

In the comparison between medulloblastoma and hemangioblastoma, the results of Mann–Whitney *U* tests suggested that GLCM-Energy (*p* = 0.022), GLCM-Homogeneity (*p* = 0.008) from T1C images, and HISTO-Skewness (*p* = 0.039), GLCM-Energy (*p* = 0.001), and GLCM-Entropy (*p* = 0.005) from FLAIR images were significantly different. There was no collinear interference observed based on the results of VIF; therefore, the above significant texture features were all introduced in binary logistic regression. The results suggested that GLCM-Energy and GLCM-Homogeneity from T1C images could be taken as independent predictors (Tables 2, 3). ROC analyses showed that the AUC of two independent predictors were 0.618 and 0.637, respectively. Standard error, 95% CI, optimal cutoff point, sensitivity, and specificity were listed in Table 4.

**TABLE 2:** Significant differences of texture features among medulloblastoma, brain metastatic tumor, and hemangioblastoma on contrast-enhanced T1-weighted images based on Mann–Whitney *U* test and binary logistic regression.

**Texture features, median (range)**	**Medulloblastoma**	**Hemangioblastoma**	**Brain metastatic tumor**	**Medulloblastoma vs. Hemangioblastoma**		**Medulloblastoma vs. Brain metastatic tumor**		**Brain metastatic tumor vs. Hemangioblastoma**	
						
				***U* test**	**Logistic regression**	***U* test**	**Logistic regression**	***U* test**	**Logistic regression**
**HISTO**									
Energy	0.034 (0.022–0.094)	0.039 (0.017–0.232)	0.029 (0.007–0.940)	0.145	–	0.389	–	0.102	–
Entropy	1.549 (1.253–1.708)	1.489 (0.787–1.774)	1.660 (0.068–2.184)	0.276	–	**0.042**	**0.001**	**0.004**	0.265
Kurtosis	3.409 (1.705–10.684)	3.121 (1.414–123.231)	2.760 (1.719–7.613)	0.884	–	**0.003**	0.084	0.081	–
Skewness	0.250 (−0.893–2.031)	0.355 (−1.055–8.167)	0.160 (−1.493–1.483)	0.503	–	0.424	–	**0.033**	**0.006**
**GLCM**									
Correlation	0.470 (−0.128–0.869)	0.436 (−0.006–0.914)	0.351 (−0.212–0.862)	0.903	–	**0.010**	**0.042**	**0.008**	0.871
Contrast	73.092 (25.181–619.718)	137.001 (6.127–447.072)	377.056 (3.039–4171.971)	0.056	–	**<0.001**	–	**<0.001**	–
Dissimilarity	6.188 (3.799–18.625)	6.919 (1.223–16.443)	13.332 (0.464–46.301)	0.884	–	**<0.001**	**<0.001**	**<0.001**	**0.001**
Energy	0.002 (0.001–0.025)	0.004 (0.001–0.101)	0.003 (0.001–0.936)	**0.022**	**0.001**	0.069	–	0.488	–
Entropy	2.776 (2.213–3.052)	2.650 (1.442–3.271)	2.739 (0.082–3.844)	0.065	–	0.716	–	0.162	–
Homogeneity	0.269 (0.169–0.465)	0.334 (0.160–0.616)	0.230 (0.097–0.973)	**0.008**	**0.009**	0.052	–	**0.003**	0.147

*HISTO, histogram-based matrix; GLCM, gray-level co-occurrence matrix. Bold values indicate statistical significance.*

**TABLE 3:** Significant differences of texture features among medulloblastoma, brain metastatic tumor, and hemangioblastoma on fluid-attenuation inversion recovery images based on Mann–Whitney *U* test and binary logistic regression.

**Texture features, median (range)**	**Medulloblastoma**	**Hemangioblastoma**	**Brain metastatic tumor**	**Medulloblastoma vs. Hemangioblastoma**		**Medulloblastoma vs. Brain metastatic tumor**		**Brain metastatic tumor vs. Hemangioblastoma**	
						
				***U* test**	**Logistic regression**	***U* test**	**Logistic regression**	***U* test**	**Logistic regression**
**HISTO**									
Energy	0.036 (0.022–0.058)	0.040 (0.022–0.104)	0.050 (0.010–0.990)	0.118	–	**<0.001**	–	**0.008**	–
Entropy	1.521 (1.339–1.681)	1.493 (1.138–1.709)	1.380 (0.030–2.120)	0.291	–	**<0.001**	–	**0.002**	–
Kurtosis	3.476 (1.968–10.271)	4.371 (1.528–25.059)	2.860 (1.630–5.690)	0.051	–	**0.002**	**0.005**	**<0.001**	**<0.001**
Skewness	−0.010 (−2.408–2.309)	0.501 (−1.406–3.143)	−0.140 (−1.350–0.920)	**0.039**	0.355	0.365	–	**0.001**	**0.045**
**GLCM**									
Correlation	0.396 (0.039–0.728)	0.383 (-0.165–0.772)	0.363 (−0.066–0.921)	0.477	–	1.000	–	0.449	–
Contrast	78.189 (26.287–196.051)	84.826 (13.586–337.520)	86.750 (2.441–2845.301)	0.477	–	0.587	–	0.667	–
Dissimilarity	6.402 (3.761–9.844)	6.383 (2.270–13.886)	5.521 (0.408–38.516)	0.916	–	0.302	–	0.365	–
Energy	0.002 (0.001–0.007)	0.003 (0.001–0.022)	0.007 (0.001–0.983)	**0.001**	0.073	**<0.001**	**0.002**	**<0.001**	**0.002**
Entropy	2.794 (2.436–3.088)	2.703 (1.927–3.102)	2.340 (0.040–3.560)	**0.005**	0.325	**<0.001**	–	**<0.001**	–
Homogeneity	0.271 (0.182–0.384)	0.282 (0.164–0.477)	0.366 (0.096–0.992)	0.257	–	**<0.001**	–	**<0.001**	–

*HISTO, histogram-based matrix; GLCM, gray-level co-occurrence matrix. Bold values indicate statistical significance.*

**TABLE 4:** The diagnostic ability of independent predictors and integrated models (*Z* score) on contrast-enhanced T1-weighted images in discrimination.

**Texture parameters**	**AUC**	**Standard error**	**95% CI**	**Optimal cutoff point**	**Sensitivity (%)**	**Specificity (%)**
**Medulloblastoma vs. Hemangioblastoma**						
GLCM-Energy	0.618	0.0547	0.528–0.702	0.005	49.23	98.41
GLCM-Homogeneity	0.637	0.0526	0.547–0.720	0.331	50.77	96.83
*Z* score	0.808	0.0380	0.729–0.872	-1.256	62.12	87.30
**Medulloblastoma vs. Brain metastatic tumor**						
HISTO-Entropy	0.607	0.0584	0.514–0.695	1.708	43.10	100.00
GLCM-Correlation	0.637	0.0506	0.544–0.722	0.382	58.62	66.67
GLCM-Dissimilarity	0.731	0.0505	0.643–0.808	13.428	50.00	98.41
*Z* score	0.825	0.0407	0.745–0.888	-1.208	67.24	95.24
**Brain metastatic tumor vs. Hemangioblastoma**						
HISTO-Skewness	0.611	0.0534	0.520–0.697	1.161	50.00	98.28
GLCM-Dissimilarity	0.723	0.0475	0.635–0.800	13.329	96.92	50.00
Z score	0.761	0.0424	0.676–0.833	1.018	93.94	50.00

*HISTO, histogram-based matrix; GLCM, gray-level co-occurrence matrix; AUC, area under the curve; CI, confidence interval.*

Moreover, the integrated model named *Z* score was built to combine the two independent predictors:

$$T\,1\,C\,i\,m\,a\,g\,e\,s:Z\,1=5.37^{*}\,G\,L\,C\,M-E\,n\,e\,r\,g\,y-1.468^{*}\,G\,L\,C\,M-H\,o\,m\,o\,g\,e\,n\,e\,i\,t\,y.$$

**FIGURE 3:**
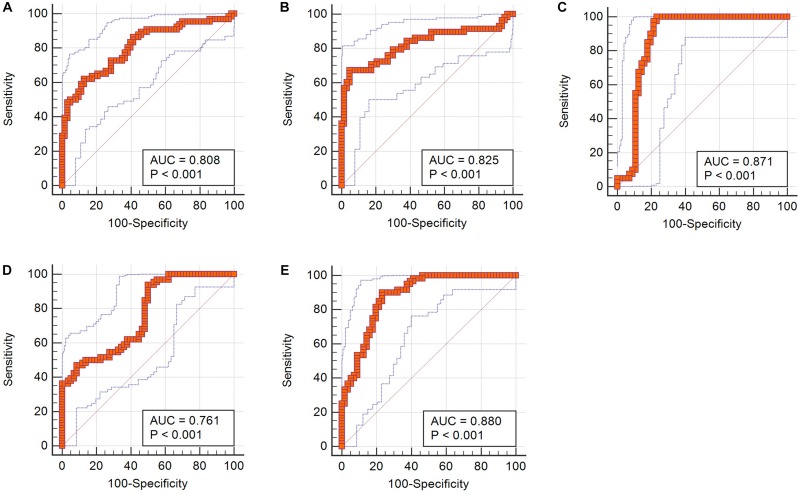
ROC curves of integrated *Z* scores in discrimination. **(A)** *Z* score from T1C images in differentiating medulloblastoma from hemangioblastoma. **(B)** *Z* score from T1C images in differentiating medulloblastoma from brain metastatic tumor. **(C)** Z score from FLAIR images in differentiating medulloblastoma from brain metastatic tumor. **(D)** *Z* score from T1C images in differentiating brain metastatic tumor from hemangioblastoma. **(E)** *Z* score from FLAIR images in differentiating brain metastatic tumor from hemangioblastoma.

Receiver operating characteristic analyses suggested that the AUC of *Z* score was 0.808, which showed much better discriminatory power than single feature and implied practical value (Figure 3).

### Part 2 (Medulloblastoma vs. Brain Metastatic Tumor)

In the comparison between medulloblastoma and brain metastatic tumor, Mann–Whitney *U* tests indicated that five texture features on T1C images [HISTO-Entropy (*p* = 0.042), HISTO-Kurtosis (*p* = 0.003), GLCM-Correlation (*p* = 0.01), GLCM-Contrast (*p* < 0.001), and GLCM-Dissimilarity (*p* < 0.001)] and six texture features on FLAIR images [HISTO-Energy (*p* < 0.001), HISTO-Entropy (*p* < 0.001), HISTO-Kurtosis (*p* = 0.002), GLCM-Energy (*p* < 0.001), GLCM-Entropy (*p* < 0.001), and GLCM-Homogeneity (*p* < 0.001)] showed significant differences. Six texture features were introduced in binary logistic regression, including HISTO-Entropy, HISTO-Kurtosis, GLCM-Correlation, and GLCM-Dissimilarity from T1C images and HISTO-Kurtosis and GLCM-Energy from FLAIR images. Binary logistic regression suggested that the above six texture features could be taken as independent predictors except for HISTO-Kurtosis on T1C images (Tables 2, 3). Detailed results of ROC analyses, including AUC, standard error, 95% CI, optimal cutoff point, sensitivity, and specificity, were summarized in Tables 4, 5.

**TABLE 5:** The diagnostic ability of independent predictors and integrated models (*Z* score) on fluid-attenuation inversion recovery images in discrimination.

**Texture parameters**	**AUC**	**Standard error**	**95% CI**	**Optimal cutoff point**	**Sensitivity (%)**	**Specificity (%)**
**Medulloblastoma vs. Brain metastatic tumor**						
HISTO-Kurtosis	0.685	0.0539	0.582–0.776	2.847	85.00	50.00
GLCM-Energy	0.808	0.0461	0.714–0.881	0.005	97.50	58.93
Z score	0.871	0.0402	0.788–0.931	76.248	100.00	76.79
**Brain metastatic tumor vs. Hemangioblastoma**						
HISTO-Kurtosis	0.757	0.0443	0.668–0.831	3.960	58.33	85.71
HISTO-Skewness	0.681	0.0511	0.588–0.764	0.445	55.00	89.29
GLCM-Energy	0.690	0.0504	0.598–0.773	0.005	73.33	58.93
Z score	0.880	0.0315	0.807–0.933	28.501	90.00	76.79

*HISTO, histogram-based matrix; GLCM, gray-level co-occurrence matrix; AUC, area under the curve; CI, confidence interval.*

Meanwhile, *Z* scores of two sequences were established to integrate the above independent predictors, respectively:

$$\mathrm{T1Cimages}:\mathrm{Z2}=-3.17^{*}\,\mathrm{HISTO}-\mathrm{Entropy}+0.714^{*}\mathrm{GLCM}-\mathrm{Correlation}+5.013^{*}\,\mathrm{GLCM}-\mathrm{Dissimilarity}.$$

$$\mathrm{FLAIRimages}:\mathrm{Z2}=1.149^{*}\,\mathrm{HISTO}-\mathrm{Kurtosis}-176.466^{*}\,\mathrm{GLCM}-\mathrm{Energy}.$$

Receiver operating characteristic analyses demonstrated that *Z* scores on both T1C images and FLAIR images could make practical value, with the AUC of 0.825 and 0.871, respectively, indicating higher diagnostic values than any single texture feature (Figure 3).

### Part 3 (Hemangioblastoma vs. Brain Metastatic Tumor)

In the comparison between hemangioblastoma and brain metastatic tumor, a total of 13 texture features were found significantly different through Mann–Whitney *U* tests: HISTO-Entropy (*p* = 0.004), HISTO-Skewness (*p* = 0.033), GLCM-Correlation (*p* = 0.008), GLCM-Contrast (*p* < 0.001), GLCM-Dissimilarity (*p* < 0.001), and GLCM-Homogeneity (*p* = 0.003) from T1C images, and HISTO-Energy (*p* = 0.008), HISTO-Entropy (*p* = 0.002), HISTO-Kurtosis (*p* < 0.001), HISTO-Skewness (*p* = 0.001), GLCM-Energy (*p* < 0.001), GLCM-Entropy (*p* < 0.001), and GLCM-Homogeneity (*p* < 0.001) from FLAIR images. After evaluating the VIF among them, HISTO-Entropy, HISTO-Skewness, GLCM-Correlation, GLCM-Dissimilarity, and GLCM-Homogeneity from T1C images, and HISTO-Kurtosis, HISTO-Skewness, and GLCM-Energy from FLAIR images were introduced in binary logistic regression, and independent predictors included HISTO-Skewness and GLCM-Dissimilarity from T1C images, and HISTO-Kurtosis, HISTO-Skewness, and GLCM-Energy from FLAIR images (Tables 2, 3). The detailed results of ROC analyses are listed in Tables 4, 5, including AUC, standard error, 95% CI, optimal cutoff point, sensitivity, and specificity.

The integrated models of two sequences were also built:

$$\mathrm{T1Cimages}:\mathrm{Z3}=-1.151^{*}\,\mathrm{HISTO}-\mathrm{Skewness}+2.127^{*}\mathrm{GLCM}-\mathrm{Dissimilarity}.$$

$$\mathrm{FLAIRimages}:\mathrm{Z3}=2.407^{*}\,\mathrm{HISTO}-\mathrm{Kurtosis}+0.646^{*}\mathrm{HISTO}-\mathrm{Skewness}-75.742^{*}\,\mathrm{GLCM}-\mathrm{Energy}.$$

In receiver operating characteristic analyses, integrated models on both T1C images and FLAIR images represented higher diagnostic values, with the AUC of 0.761 and 0.880, respectively (Figure 3).

### Prediction for Three Tumors

Considering the relatively limited number of FLAIR images, multinomial logistic regression was only conducted on texture features extracted from T1C sequence. In accordance with the results of above pairwise comparisons, HISTO-Entropy (*p* = 0.011), HISTO-Kurtosis (*p* = 0.026), GLCM-Correlation (*p* = 0.011), GLCM-Contrast (*p* < 0.001), GLCM-Dissimilarity (*p* < 0.001), GLCM-Energy (*p* = 0.048), and GLCM-Homogeneity (*p* = 0.002) from T1C images showed significant difference based on Kruskal–Wallis *H* test (Supplementary Table S1). With brain metastatic tumor as the referent category, two multinomial logistic regression models were established: radiomic predictive model included the seven significant texture features; comprehensive predictive model added the age of patient (Table 6). The regression equation of the two models was listed as follows:

**TABLE 6:** Regression coefficients and significance levels for each variable in two multinomial logistic regression models with brain metastatic tumor as the referent category.

**Parameters**	**Medulloblastoma vs. Brain metastatic tumor**		**Hemangioblastoma vs. Brain metastatic tumor**	
		
	***B***	***p* value**	***B***	***p* value**
**Radiomic predictive model**				
HISTO-Entropy	0.583	0.893	–3.715	0.106
HISTO-Kurtosis	0.250	0.076	0.252	0.058
GLCM-Correlation	–0.374	0.853	2.353	0.092
GLCM-Contrast	–0.016	0.282	–0.017	0.020
GLCM-Dissimilarity	0.187	0.805	0.808	0.041
GLCM-Energy	–232.413	0.084	–10.365	0.385
GLCM-Homogeneity	0.214	0.984	2.000	0.654
Intercept	0.617	0.787	–0.157	0.952
**Comprehensive predictive model**				
Age	–0.302	<0.001	–0.046	0.006
HISTO-Entropy	–6.429	0.237	–4.797	0.044
HISTO-Kurtosis	–0.175	0.614	0.161	0.128
GLCM-Correlation	0.273	0.933	2.469	0.071
GLCM-Contrast	–0.057	0.027	–0.023	0.011
GLCM-Dissimilarity	2.326	0.047	1.079	0.018
GLCM-Energy	8.213	0.773	–12.517	0.386
GLCM-Homogeneity	–5.871	0.590	3.677	0.428
Intercept	10.967	0.025	2.406	0.267

*HISTO, histogram-based matrix; GLCM, gray-level co-occurrence matrix. *B*, regression coefficient for each variable.*

Radiomic predictive model:

$$\mathrm{Logit}\,\left(\mathrm{p1}/\mathrm{p3}\right)=0.617+0.583^{*}\,\mathrm{HISTO}-\mathrm{Entropy}+0.250^{*}$$

$$\mathrm{HISTO}-\mathrm{Kurtosis}-0.374^{*}\,\mathrm{GLCM}-\mathrm{Correlation}-0.016^{*}$$

$$\mathrm{GLCM}-\mathrm{Contrast}+0.187^{*}\,\mathrm{GLCM}-\mathrm{Dissimilarity}-232.413^{*}$$

$$\mathrm{GLCM}-\mathrm{Energy}+0.214^{*}\,\mathrm{GLCM}-\mathrm{Homogeneity}.$$

$$\mathrm{Logit}\,\left(\mathrm{p2}/\mathrm{p3}\right)=-0.157-3.715^{*}\,\mathrm{HISTO}-\mathrm{Entropy}+0.252^{*}$$

$$\mathrm{HISTO}-\mathrm{Kurtosis}+2.353^{*}\,\mathrm{GLCM}-\mathrm{Correlation}-0.017^{*}$$

$$\mathrm{GLCM}-\mathrm{Contrast}+0.808^{*}\,\mathrm{GLCM}-\mathrm{Dissimilarity}-10.365^{*}$$

$$\mathrm{GLCM}-\mathrm{Energy}+2.000^{*}\,\mathrm{GLCM}-\mathrm{Homogeneity}.$$

Comprehensive predictive model:

$$\mathrm{Logit}\,\left(\mathrm{p1}/\mathrm{p3}\right)=10.967-0.302^{*}\,\mathrm{Age}-6.429^{*}$$

$$\mathrm{HISTO}-\mathrm{Entropy}-0.175^{*}\,\mathrm{HISTO}-\mathrm{Kurtosis}+0.273^{*}$$

$$\mathrm{GLCM}-\mathrm{Correlation}-0.057^{*}\,\mathrm{GLCM}-\mathrm{Contrast}+2.326^{*}$$

$$\mathrm{GLCM}-\mathrm{Dissimilarity}+8.213^{*}\,\mathrm{GLCM}-\mathrm{Energy}$$

$$-5.871^{*}\,\mathrm{GLCM}-\mathrm{Homogeneity}.$$

$$\mathrm{Logit}\,\left(\mathrm{p2}/\mathrm{p3}\right)=2.406-0.046^{*}\,\mathrm{Age}-4.797^{*}\,\mathrm{HISTO}-\mathrm{Entropy}$$

$$+0.161^{*}\,\mathrm{HISTO}-\mathrm{Kurtosis}+2.469^{*}\,\mathrm{GLCM}-\mathrm{Correlation}$$

$$-0.023^{*}\,\mathrm{GLCM}-\mathrm{Contrast}+1.079^{*}\,\mathrm{GLCM}-\mathrm{Dissimilarity}$$

$$-12.517^{*}\,\mathrm{GLCM}-\mathrm{Energy}+3.677^{*}\,\mathrm{GLCM}-\mathrm{Homogeneity}.$$

(p1, p2, and p3 were the predictive possibility of medulloblastoma, hemangioblastoma, and brain metastatic tumor, respectively.) Compared with the radiologist (accuracy = 0.72), both models represented feasible ability in differentiation, with an accuracy of 0.69 in radiomic predictive model and 0.80 in comprehensive predictive model (Table 7).

**TABLE 7:** Comparison of accuracy in predicting the three types of posterior fossa tumors among radiomic predictive model, comprehensive predictive model and radiologist.

	**Radiomic predictive model**	**Comprehensive predictive model**	**Radiologist**
Accuracy	0.69	0.80	0.72

## Discussion

In the current study, we investigated the diagnostic value of texture analysis on MRI in differentiating the three most common posterior fossa tumors. Texture features from T1C images and FLAIR images were found to be significantly different, and the integrated models represented promising discriminatory performance. Moreover, we combined radiomics and clinical parameters to establish the diagnostic model that showed feasible ability in prediction and could potentially be served as an ancillary tool to aid radiological diagnosis.

It is important to discriminate among medulloblastoma, hemangioblastoma, and brain metastatic tumor due to their dramatically different clinical management. For hemangioblastoma, the standard therapy is complete surgical resection; for medulloblastoma, the first-line therapy is maximal safe resection followed by postoperative radiotherapy and possibly adjuvant chemotherapy; for brain metastatic tumor, the treatment strategy could be surgical resection, radiosurgery, radiotherapy, chemotherapy, or a combination of these, based on the size, location, and primary origin of the tumor. Thus, a non-invasive and practical diagnostic method is necessary and could be beneficial for treatment planning and discussion with the patient.

Texture analysis, the mathematical method for the quantitative analysis of the variation in image patterns, had been proven to show promising diagnostic potential in various brain tumors, like glioma, meningioma, brain metastatic tumor, primary central nervous system lymphoma, and medulloblastoma (Mouthuy et al., 2012; Rodriguez Gutierrez et al., 2014; Suh et al., 2018; Xiao et al., 2018; Lu et al., 2019). One study demonstrated that three-dimensional texture analysis on MRI could be used in discriminating glioblastoma from primary central nervous system lymphoma (Xiao et al., 2018). Another study investigated the value of various texture features in distinguishing brain metastatic tumor from high-grade glioma (Mouthuy et al., 2012). Similar to our results, they reported that GLCM-Energy, a parameter that reflected uniformity of gray-level voxel pairs, was statistically significant in discrimination. Besides, one study applied texture analysis in distinguishing medulloblastoma from pilocytic astrocytoma and ependymoma, suggesting that several texture features from diffusion-weighted imaging in HISTO matrix potentially represented promising diagnostics (Rodriguez Gutierrez et al., 2014). However, the value of texture analysis in differentiation among posterior fossa tumors was still unclear. Thus, we applied texture analysis in three of the most common tumors in this region, including medulloblastoma, hemangioblastoma, and brain metastatic tumor. Encouragingly, several texture features from T1C images and FLAIR images represented significant differences among the three types of tumors. Although the diagnostic value of single texture feature was relatively limited, the integration of independent texture features displayed practical discriminatory ability with an AUC of more than 0.800 in all pairwise comparisons. Therefore, texture analysis on conventional MRI could be potentially recognized as a valuable method to aid diagnosis of these tumors by providing additional quantitative information.

To our best knowledge, texture analysis had never been performed in the discrimination among the three common posterior fossa tumors, and the integration of texture features was rarely seen in previous studies. As mentioned above, the texture feature could reflect the characteristics of MR images from various aspects, such as asymmetry of the gray-level distribution, variation of gray-level voxel pairs, or uniformity of gray-level voxel pairs. Therefore, by combining single texture feature through established formula, the integrated *Z* score could depict the radiological characteristics of the tumor more systematically. This might explain the reason why the integrated model represented the higher diagnostic value than any single texture feature. Moreover, considering the age of the patient was an important clinical factor in differentiating the three types of tumors, we combined the clinical and radiomics parameters to build the comprehensive predictive models with higher accuracy than radiomic predictive model that only included texture features. More importantly, compared with radiologists, the comprehensive model represented comparable or even better performance in prediction.

The relationship between texture features and characteristics of the tumor was complicated. A previous study had implied that texture features derived from GLCM might be associated with the heterogeneity of tumors, which could be reflected in the MR images (Lakhman et al., 2017). Brain metastatic tumor has usually been observed to be heterogeneous in MR images arisen from the necrosis, cyst formation, or hemorrhage within the tumor. Medulloblastoma typically appears as a heterogeneous mass due to intratumoral cystic components or calcification. Hemangioblastoma with cystic cavity could also show heterogeneous signal intensity. Such differences of heterogeneity among the three types of tumors could be quantitatively observed in the GLCM-derived texture features (Tables 2, 3). Another study on head and neck tumor indicated that texture features could also reflect the tissue density within the tumor (Fujima et al., 2019). Besides, GLCM-Correlation, which measures linear dependency of gray levels, was found to be linked to the vascularity of lesion (Nardone et al., 2018). However, unanimous conclusions were not reached since texture features were derived from different matrixes on various types of MR images, and the relationship between texture features was complicated. More researches are required to explore the specific connection between the tumor biology and texture parameters.

There were several limitations in the present study. First, it was a retrospective study, and only patients with preoperative MR scan were enrolled, bringing the selection bias inevitably. Second, we only assessed the value of texture analysis on T1C images and FLAIR images. Third, considering the limited number of patients, we did not conduct texture analysis among the subtypes of tumors as inadequate sample size made logistic regression unfeasible. The diagnostic value of texture analysis in discriminating molecular subtypes of medulloblastoma and brain metastatic tumor originated from different organs required more research in the future. Fourth, we only analyzed texture features derived from HISTO and GLCM since they were two of the most common matrixes in previous studies and were reported to be associated with the underlying histopathological characteristics of the tumor. Future studies with larger sample size were required to validate our results and explore the value of texture features from other matrixes.

## Conclusion

Magnetic resonance imaging -based texture analysis had the potential to be served as a feasible method in discriminating the three most common posterior fossa tumors including medulloblastoma, brain metastatic tumor, and hemangioblastoma. Several texture features from T1C images and FLAIR images showed significant difference, and the integration of them displayed better discriminative performance. Moreover, a comprehensive predictive model that integrated texture features and the age of patient could potentially be applied to predict the three types of tumors with practical value.

## Data Availability Statement

The data used to support the findings of this study are available from the corresponding author upon request.

## Ethics Statement

All procedures performed in studies involving human participants were in accordance with the ethical standards of the institutional and/or national research committee and with the 1964 Helsinki declaration and its later amendments or comparable ethical standards. Informed consent was obtained from all individual participants included in the study. This study was approved by the institutional ethics review board.

## Author Contributions

YZ: conception of the study, draft writing, and manuscript revision. CC: data collection and manuscript revision. ZT: data collection and statistical analysis. RF: data analysis and interpretation. YC: data collection. JX: conception of the study and manuscript revision. All authors read and approved the submitted version.

## Conflict of Interest

The authors declare that the research was conducted in the absence of any commercial or financial relationships that could be construed as a potential conflict of interest.

## Footnotes

1 http://www.lifexsoft.org
