# Supplementary material for: The Diagnostic Value of MRI-Based Texture Analysis in Discrimination of Tumors Located in Posterior Fossa: A Preliminary Study
Source: Front Neurosci. 2019 Oct 23;13:1113. doi: 10.3389/fnins.2019.01113 (PMC6819318; doi:10.3389/fnins.2019.01113)
Supplement: Supplementary file 1 [file Table_1.DOCX]

**Supplement Table 1.** Differences of texture features among medulloblastoma, brain metastasis and hemangioblastoma on contrast-enhanced T1-weighted image based on Kruskal-Wallis H test.

| Texture feature, median (range) | Medulloblastoma | Hemangioblastoma | Brain metastasis | Chi-Square | p-value |
| --- | --- | --- | --- | --- | --- |
| HISTO |  |  |  |  |  |
| Energy | 0.034 (0.022~0.094) | 0.039 (0.017~0.232) | 0.029 (0.007~0.940) | 3.633 | 0.163 |
| Entropy | 1.549 (1.253~1.708) | 1.489 (0.787~1.774) | 1.660 (0.068~2.184) | 9.023 | **0.011** |
| Kurtosis | 3.409 (1.705~10.684) | 3.121 (1.414~123.231) | 2.760 (1.719~7.613) | 7.276 | **0.026** |
| Skewness | 0.250 (-0.893~2.031) | 0.355 (-1.055~8.167) | 0.160 (-1.493~1.483) | 5.961 | 0.051 |
| GLCM |  |  |  |  |  |
| Correlation | 0.470 (-0.128~0.869) | 0.436 (-0.006~0.914) | 0.351 (-0.212~0.862) | 9.074 | **0.011** |
| Contrast | 73.092 (25.181~619.718) | 137.001 (6.127~447.072) | 377.056 (3.039~4171.971) | 28.895 | **<0.001** |
| Dissimilarity | 6.188 (3.799~18.625) | 6.919 (1.223~16.443) | 13.332 (0.464~46.301) | 24.574 | **<0.001** |
| Energy | 0.002 (0.001~0.025) | 0.004 (0.001~0.101) | 0.003 (0.001~0.936) | 6.087 | **0.048** |
| Entropy | 2.776 (2.213~3.052) | 2.650 (1.442~3.271) | 2.739 (0.082~3.844) | 3.723 | 0.155 |
| Homogeneity | 0.269 (0.169~0.465) | 0.334 (0.160~0.616) | 0.230 (0.097~0.973) | 12.471 | **0.002** |

Abbreviations: HISTO, Histogram based matrix; GLCM, Grey-level co-occurrence matrix.
